# Treatment of Chinese adolescents with anorexia nervosa in Hong Kong: The gap between treatment expectations and outcomes

**DOI:** 10.1371/journal.pone.0216582

**Published:** 2019-05-09

**Authors:** Kai Sing Sun, Tai Pong Lam, Kit Wing Kwok, King Yee Chong, Man Kay Poon, Dan Wu

**Affiliations:** 1 Department of Family Medicine and Primary Care, The University of Hong Kong, Hong Kong, China; 2 Department of Psychiatry, The University of Hong Kong, Hong Kong, China; 3 Faculty of Infectious and Tropical Diseases, London School of Hygiene and Tropical Medicine, London, United Kingdom; International Telematic University Uninettuno, ITALY

## Abstract

**Background and objective:**

Anorexia nervosa (AN) is one of the most difficult-to-treat psychiatric disorders. AN is associated with individual vulnerability, societal and family factors. There has been limited research in Asia regarding the patients or their families’ perceptions on its treatment. This study explored the perceived treatment outcomes among Chinese families having adolescents with AN.

**Methods:**

Qualitative interviews were conducted on parents of adolescents with AN recruited through an eating disorder association in Hong Kong to understand their views and experiences regarding the help-seeking and treatment process.

**Results:**

The parents expressed dissatisfaction towards help-seeking and treatment outcomes, including relationships with health professionals, hospitalization, health professionals’ knowledge of AN, understanding of the treatment model and parents’ role, amount of psychological support, and coordination among health professionals. The parents were unclear about the treatment plan as they received little explanation from the health professionals. The parents perceived that the AN treatment only focused on weight restoration with limited psychological support. Home diet monitoring was seen as a harsh task which worsened the relationship with their children. The parents often needed to take up the coordinator role and search around for different health professionals and integrate their advices by themselves.

**Conclusions:**

The study shows that limited psychoeducation, communication and coordination in the treatment for AN are major problems in a Chinese context. Open communication between the health professionals and the parents about the expected treatment outcomes and limitations is needed to enhance their mutual trust. Besides, treatment should emphasize not only family involvement but also empower them to fight against AN.

## Introduction

Eating disorder (ED) is classified into three types including: anorexia nervosa (AN), bulimia nervosa, and binge eating disorder. Considered as one of the most difficult-to-treat psychiatric disorders, AN often results in poor treatment outcomes [1]. According to DSM-5, AN is diagnosed with the symptoms of severely restricted energy intake resulting in abnormally low body weight, intense fear of weight gain, excessive weight and shape concerns [2, 3]. Apart from diet restriction, some AN patients have purging behaviours [4]. Pubertal development increases vulnerability to AN in adolescence [5] and nearly 90% of AN patients are female [6]. In high-income countries, the point prevalence of AN was reported to be 0.3–0.5% [4], and the incidence was high among females aged 15–19 (109.2 per 100 000) [7]. Epidemiological studies in East Asia revealed a rise in the prevalence of AN across the territories. AN prevalence in Japan had increased from 0.11% to 0.43% in 20 years [8] and was found to be comparable to the estimates of North America and Europe [9]. A recent study in Wuhan, China found that the prevalence of ED among female university students was even higher than that of Western countries [10]. Although there was little information in Hong Kong that described the local prevalence of AN, a study found that 5.1% of adolescents aged 10–21 had disordered eating behaviours [11].

Past studies suggested that AN was associated with individual vulnerability including genetic factors, neurobiological factors, co-morbid conditions of obsessive-compulsive disorder and personality disorders [4, 12–14]. Moreover, there were also external risk factors of AN during adolescence, including appearance-based social pressure from parents, peers, and/or mass media, weight and body dissatisfaction and maladaptive family functioning [12, 15, 16]. Increased demand for autonomy and growing relationship with peers in early adolescence might threaten family organization and functioning. It was found that adolescents with AN perceived their family as highly disengaged, poorly interwoven, overly protective and rigid, with low family cohesion and communication qualities [12, 17]. Food refusal was also found as a form of revenge to the parents’ control and a protest to express the need for autonomy, especially in a Chinese context [6]. Updated treatment protocols emphasized principal outcomes not only in terms of physical impairment but also psychological distress, social functioning (with families and peer) and psychopathological comorbidities [4, 18]. A recent review found that psychotherapies especially family-based treatment (FBT) was recommended in the clinical guidelines of seven Western countries, while six countries recommended cognitive-behavioural-therapy (CBT) for individual psychotherapy, and one for adolescent-focused therapy (focused on autonomy and self-efficacy) [19]. FBT, an outpatient-based treatment that emphasized parental involvement to support their children for behavioural changes, offered the strongest level of evidence for treating adolescent AN, whereas CBT, adolescent-focused therapy and systematic family therapy (similar to FBT, but focused on family system and interactions [20]) only had moderate level of evidence [4]. Evolving from the earlier view that family problems or parenting issues are the underlying causes of AN, FBT adopts a more positive perspective to empower and engage the parents in the management of their children’s AN [21]. The parents take on an important role in ensuring weight restoration at home during the initial phase of treatment, before returning control over eating to the children in phase 2 and establishing a healthy adolescent relationship with the parents in phase 3 [13, 22].

Although researches provided evidence on the effectiveness of treatment, mainly by FBT and its variants, less than half of the patients remained weight-restored in the long run [23]. By investigating patients’ perspectives towards treatment, studies in the West identified several factors that might have limited the success of treatment. Patients perceived professional assistance at both inpatient ward and outpatient clinics as unhelpful because their behaviours had not modified after treatments [24, 25], and the standardized program also failed to cater for individual differences [26]. Some patients felt that the treatments emphasized too much on weight management but paid insufficient attention to their emotional needs [26, 27]. The lack of empathy for patients reflected a lack of knowledge on the professionals’ part because they were not trained to be specialized in treating ED [28]. While it was unclear whether FBT had been applied, patients with severe conditions needed to be hospitalized where strict behavioural approaches were adopted for treatment. These approaches were criticized by patients for their inflexibility and award/punishing nature, thus accounting for the negative perception from patients of their effectiveness and sustainability [26, 29].

In Asia, limited studies have been conducted to investigate the perceptions of ED patients towards treatment, though there were a few studies that mainly evaluated the treatment effectiveness of FBT. A qualitative study in Hong Kong found that FBT helped to reduce family conflicts and resolved the problem of self-starvation [18], while another study highlighted that trust between the patient and the therapist was crucial in treating AN [30]. Although findings suggested that FBT might be effective in treating AN in a Chinese context, it has not been widely adopted in Hong Kong. Owing to limited manpower and resources, the child and adolescent psychiatric services offered in public hospitals are still dominated by a biomedical model [31].

This study is part of a larger project investigating barriers and enablers to help-seeking for mental health problems in Hong Kong. Findings regarding psychological distress have been published elsewhere [32–34]. Apart from adult patients, significant others of both adult and child patients were interviewed. We found distinct treatment barriers for families having adolescents with AN through qualitative interviews. This paper analyses the help-seeking and treatment outcomes for AN among a sample of families. The findings may help to improve health services and enhance support to the families encountering AN.

## Methods

### Sources of participants

A qualitative study on six families having female adolescents with AN were conducted. The parents were invited for interview through an association of eating disorder in Hong Kong. The association is a non-profit organization set up by ED patients and their family members, with a professional advisory team of psychiatrists, family doctors, psychologists, social workers and dietitians. Six parents, consisting of four females and two males, participated in the in-depth interview and discussion. Their ages ranged from 49–57 years; four of them had secondary education level and two were tertiary educated (details shown in Table 1). Ethics approval was obtained from the Institutional Review Board of The University of Hong Kong /Hospital Authority Hong Kong West Cluster (UW 09–326). Written informed consent was obtained from the participants.

**Table 1:** Case summaries.

#	Sex	Age	Education	Case description
P1	F	50	Secondary	Consulted clinical psychologist from the staff clinic where P1 worked first but not diagnosed with AN. As P1’s daughter kept losing weight, they then consulted a general practitioner in the public clinic. Her daughter was diagnosed with AN and was referred to a psychiatrist. The waiting time was too long that they finally went to the accident and emergency department (AED) where her daughter was handled by the in-house psychiatrist who was available on specific dates. They had a negative experience with the psychiatrist and P1 was distressed about the tough role of parents in enforcing food intake.
P2	M	50	Tertiary	P2 had two daughters with AN. They consulted a neurologist at the beginning and were referred to see the psychiatrist. They had also consulted different psychiatrists, clinical psychologists, family therapists, nutritionists and paediatricians. The perceived treatment outcomes were mostly ineffective and P2 thought there was a lack of treatment alternatives in the local setting. His daughters were attending hospital out-patient clinics for regular physical check-ups.
P3	M	49	Secondary	A school social worker told P3 that his daughter had anorexia. P3 regarded it as a physical illness and took his daughter to AED. His daughter was then referred to psychiatrists/ psychologists. The treatment was useless in his eyes. Instead, his daughter stayed in the paediatrics department and the physical treatment was quite effective to increase her weight. As recommended by another AN patient in the ward, they joined the eating disorders association. The successful experience sharing from other patients helped a lot to cure his daughter. Besides, support from the school social worker also strengthened her self-esteem and assisted the recovery process.
P4	F	57	Secondary	Consulted a TCM practitioner who claimed that herbal tea could cure the problem. As her daughter’s conditions got worse, they saw psychiatrists at a hospital and were prescribed drugs. However, her daughter did not want to rely on drugs and she stopped taking them. She was then followed-up by a clinical psychologist but stopped the treatment later, as P4 felt the clinical psychologist was a mediator who actually blocked her direct communication with her daughter.
P5	F	53	Secondary	P5’s daughter had AN. Since her daughter menstrual period stopped, they sought help from a gynaecologist and then a neurologist. Although interpreted as a stress related problem, the doctors felt she would recover later. After several years, the daughter’s AN progressed into BN and a lot of conflicts arose between her and her daughter. Hence they went see a social workers and her daughter was referred to the hospital’s psychiatry department. She emphasized the importance of the role of family in recovery.
P6	F	54	Tertiary	The family doctor referred her daughter to a gastroenterologist to perform costly tests but he was unable to identify the problem. A school teacher suspected that P6’s daughter might had anorexia and P6 soon forced her daughter to visit AED as her conditions got worse. This made her daughter angry and she ran away from home for a short time. Her daughter was treated by psychiatrists, clinical psychologists, paediatricians and nutritionists. However, she finally decided to stop the treatments as she and her daughter did not trust the specialists.

### Data collection

Employing the form of a semi-structured interview in a group format, we asked open ended questions (“what”, “how” and “why”) to enquire about the parents’ views and experience on the barriers and enablers of help-seeking, treatment and recovery for their children’s AN. Instead of hypothesis testing or starting from a fixed framework, we adopted a grounded theory method which was an inductive approach to derive patterns, themes, and common categories based on the cases shared by the participants [35]. We aimed to avoid pre-assumption of the attitudes of the participants. The two interviewers are researchers experienced in qualitative and mental health studies, but not practicing medical doctors or therapists. They held a neutral stance and did not judge the views of the parents. Each parent shared the detailed story of his/her daughter’s case. The two interviewers asked open ended questions to guide the case sharing and invite opinions from other parents to enable interactions and feedback. The interaction dynamics accentuated participants' similarities and differences and offered a range of views on the themes.

### Analysis

The interviews were conducted in Cantonese and audio-recorded, and then were transcribed verbatim. The accuracy of the transcripts was checked against the audio recordings by an investigator. Using the content analysis approach described by Hsieh and Shannon [36], coding categories were inductively derived from the text data. The data were coded independently by two investigators of the research team who are experienced in qualitative research. The coding consistency between the two sets was checked and most of the codes were consistent. Inconsistencies were resolved by discussion between the two investigators to reach an agreement for a common theme. The views and experiences of the respondents on help-seeking, treatment and recovery for AN were identified and analysed under different themes. Selected quotes from the participants were translated into English for the write-up of this article.

## Results

### Overall help seeking pattern

The case summaries are shown in Table 1. The physical symptoms such as weight loss and amenorrhea triggered the parents to bring their children to see the doctors. Apart from the parents, school social workers might also notice the problems and alert the parents. Before approaching the psychiatric departments, most patients had consulted general practitioners (GPs) or doctors in specialty other than psychiatry, such as gastroenterologists, gynecologists and neurologists. Some with severe weight loss had visited the accident and emergency department (AED). They were then referred to other health professionals for AN treatment, including psychiatrists, clinical psychologists, pediatricians and nutritionists.

### Barriers

The parents described strong barriers in the help-seeking and treatment process, ranging from relationships with health professionals, hospitalization, knowledge of AN among health professionals, understanding of the treatment model, the role of parents in diet management at home, amount of psychological support, to coordination among health professionals. The themes of barriers and relevant quotes are summarized in Table 2.

**Table 2:** Barriers in the help-seeking and treatment process.

Themes		Quotes
Perceived limited empathy from doctors	1	During the consultation, we were scolded by the psychiatrist [in A & E]. Have I done anything wrong? He told me that my daughter was well-behaved but I left her in other people’s care. Hey, I have to work! I have already tried my best to find something that is suitable for my daughter…The psychiatrist thought that we threw our daughter off our care over and over again as if she was a ball…..He thought that we didn’t care about our daughter. (P1)
	2	The psychiatrist asked my twelve-year-old daughter if she would like to go to Castle Peak Hospital (a local psychiatric hospital). My daughter didn’t respond to him. Since she had no response, he said that there should be no problem. I was not very contented and wondered if he had a problem. What did you expect her to answer? She was just twelve years old! (P1)
Fear of admission to psychiatric hospitals	3	We once went to the psychiatric hospital to have a look. We felt upset afterwards as we saw a lot of bars at the windows and it looked like a prison. It was terrible. I was concerned as my daughter was little, and patients were grouped into the same ward despite difference in age and the mental disorders they had. (P1)
	4	She refused to see the doctor as she could still clearly recall the experience of seeing the doctor when she was 11 years old. She did not want to be admitted to the hospital compulsorily again. She even refused to visit the doctor when she had a common cold. (P6)
Health professionals’ limited knowledge on AN	5	Perhaps it was just a common gynecological disease. The doctor told us not to worry as she could have missed her periods because of stress. She ended up with bulimia at a later stage, but remained skinny despite the fact that she had been eating frequently. Again, the doctor told us not to worry and let her eat whatever she liked as she looked thin. Perhaps we were still unfamiliar with the disease at that time… (P5)
	6	We consulted the third [private] psychiatrist for second opinion, and he recommended us to send my daughter to hospital. We felt that he didn’t want to treat anorexia patients. Maybe it’s because one could die from anorexia, and the psychiatrist did not have much experience in managing anorexia… Most of them are not willing to treat child patients. They would ask us not to consult them again and would keep their doors shut. (P2)
	7	I think the nutritionists were quite unfamiliar with the disease [AN] too. They only asked her to eat because she looked skinny. The social worker, I mean the one in the hospital, knew little about the disease. She told me that I should stop letting my daughter have snacks. I was also being asked to encourage her to take proper meals and refrain from overeating. (P5)
Limited explanation of the treatment model	8	The [psychiatric] hospitals are well-equipped. However, we found that they [psychiatrists] had done little to treat her. They just had brief chats with her. They did not tell you how they would treat her. What I mean is that they had never told me that they had a treatment plan for her…. I felt the treatment she received was completely useless (P2)
	9	I also think that most psychiatric consultations are not helpful. The psychiatrists repeated their questions every time [when] we visited [again] and what they said were not constructive [to my daughter]. (P6)
	10	Doctors in hospital mainly forced her to eat. If she could reach a certain weight, she could be discharged from the hospital. That’s it. (P4)
Insufficient communication between parents and health professionals	11	I thought it was quite good at first, as I could at least obtain some information about my daughter [from the clinical psychologist]. However, later, I realized that there was a problem. For example, as my daughter visited the clinical psychologist biweekly, she would not tell me anything that happened within the two weeks. My daughter would only reveal them to the psychologist during the consultation, and the psychologist would talk to me about her experiences. I felt like I had to rely on a third person to communicate with my daughter. (P4)
	12	The psychologist was also unhelpful. Why? I usually meet him before my daughter during a consultation. He told my daughter that he would tell me what he had discussed with her during our subsequent visit. However, he told me nothing about it in our subsequent visit. (P6)
Inadequate understanding of parents’ role in managing AN	13	The story happened twelve to thirteen years ago. The psychiatrist thought that some children could be forced, which meant I could also force my daughter to eat. But there are some children that can’t be forced. Anyway, I tried to force my daughter according to what the psychiatrist said, but it wasn’t very effective. Normally, parents would not interfere with the eating habits of their children, but I had to… He asked my daughter to record down what she had for breakfast, lunch and dinner. Since my daughter refused to write them down, I did it for her. I thought I was involved in this matter as I had to recall and record down what she ate. I even had to weight the food … The relationship between my daughter and I worsened as the doctors forced us to monitor her while she was eating. Our relationship remained poor for a very long time. (P1)
	14	I think their regulations are relatively loose. I understand that she is cunning sometimes. As they [the psychiatrists] only allowed her to go to school if her weight reached a particular standard, my daughter would hold her urine, put several locks into her pockets and drink a lot of water [to increase the apparent body weight]. At first, they weren’t aware of it. Later, they explained to me that they didn’t let her go to school as a lock fell from her pocket when they were measuring her weight. Then I said “What?” Sometimes, I was doubtful about it. You wanted me to be strict, but your regulations were loose. I also thought that the psychiatrist had made me become a villain…Actually, should the psychiatrist take up this role instead? (P1)
Insufficient coordination among health professionals	15	If I try to consult all types of healthcare professionals, including a psychiatrist, a psychologist, a medical doctor and a family therapist, then I would have to explain everything from beginning when I meet each of them… And then what they said [about my daughter] were inconsistent. Then, I would have to… As parents, it is difficult [for me] to coordinate [the work]. We are not professionals and we lack the knowledge [of the disease]. (P2)
Insufficient treatment options	16	I read a US book about anorexia and found that the doctor in the book had been treating anorexia for 40 years. Therefore, I emailed him and asked for his help…We used Skype, but this method didn’t work very well because of the distance. (P2)
	17	There are several types of psychological therapies. They could be individually based, family-based and group-based. However, I really don’t know where you could find family therapists that specialize in treating anorexia in Hong Kong… In foreign countries, there is usually a team which put strong emphasis on family support and teamwork, and such kind of support is totally unavailable in Hong Kong. (P2)

#### Perceived limited empathy from doctors

Limited empathy and lack of support from doctors to caregivers were revealed. Negative comments from the psychiatrists about the parental approach aroused bad feelings in some of the parents. P1 was upset as the psychiatrist told her that she should not have left her daughter in other people’s care, but she thought she had already tried her best to take care of her daughter despite of her busy schedule (Table 2, Quote 1). P1 was also dissatisfied with the communication style of the psychiatrist with her daughter (Quote 2).

#### Fear of admission to psychiatric hospitals

The parents were worried about placing their children in a psychiatric hospital, though it was a usual management approach for severe cases. They were concerned about the quarantined environment in psychiatric wards and disliked the thought that their children had to mix with patients suffered from severe psychiatric disorders. They were also worried that the in-patient medical record might affect their children’s future career and medical insurance. The negative experiences associated with previous hospitalization discouraged P6’s daughter from seeking medical help again (Quotes 3 and 4).

#### Health professionals’ limited knowledge on AN

Previous help seeking experiences revealed inadequate knowledge on AN among health professionals to recognize the disorder. Parents felt that doctors not being psychiatrists generally lacked understanding of AN and were unable to offer helpful treatments (Quote 5). P2 felt that, even among psychiatrists or clinical psychologists, many were unwilling to manage adolescent AN patients because they might lack sufficient knowledge and experience to treat the condition well (Quote 6). Similarly, the parents felt that social workers and nutritionists were also not knowledgeable enough to offer useful advices on AN (Quote 7).

#### Limited explanation of the treatment approach

The parents had little knowledge or information about the treatment approach or model that the psychiatrists specialized in treating AN had employed to treat their children. They were unclear whether FBT or other common models were applied. Moreover, the parents were not satisfied with the little amount of counselling and patient education provided by the psychiatrists. P2 felt the psychiatrists did not explain well the treatment plan and the cause of the illness (Quote 8). P6 added that some psychiatrists just repeated their words in the follow-up sessions. They usually focused on increasing the body weight of the patients by behavioural control rather than offering them psychological and family support (Quotes 9 and 10).

#### Insufficient communication between parents and health professionals

Respondents also negatively commented on the experiences they had with clinical psychologists. The clinical psychologists by job nature provided psychological counselling. While the communication between the clinical psychologists and the children tended to be good, it might not be the case regarding communication with the parents (Quotes 11 and 12).

#### Inadequate understanding of parents’ role in managing AN

Limited understanding of the treatment model and communication with health professionals led to little understanding of the parents’ role to the success of treatment. The parents perceived that they needed to take the harsh role in monitoring their children’s eating behaviour at home and this often worsened their family relationships (Quote 13). P1 was angry that the doctors asked the parents to be strict in their child’s diet management for weight restoration but the hospital staff themselves did not strictly follow the treatment protocol and outcome assessment (Quote 14).

#### Insufficient coordination among health professionals

There were often different views between psychiatrists and clinical psychologists on the management approaches of parents. The discrepancies confused the parents. P2 felt that there was insufficient coordination between health professionals involved because they did not work as a team approach like those in Western countries. P2 needed to take up the coordination role himself and described the case again and again (Quote 15).

#### Insufficient treatment options

When the parents were disappointed with the usual treatment approaches such as weight restoration and monitoring, they looked for alternatives. P2 attempted to contact a therapist in the US who advocated a treatment strategy which engaged AN patients in role playing. However, the treatment was unavailable in public medical services in Hong Kong. His daughter thus received an alternative treatment through Skype, but the outcome was poor due to the communication barriers (Quote 16). P2 also thought there was a lack of FBT in the local public healthcare setting (Quote 17).

### Enablers

While the parents tended to share negative experiences, some enablers to treatment were also revealed. Hospitalization in pediatric wards, empathetic healthcare professionals together with support from patient organizations and schools were useful to encourage help-seeking bahaviors. The themes of enablers and relevant quotes are summarized in Table 3.

**Table 3:** Enablers in the help-seeking and treatment process, and reflections.

Themes		Quotes
Inpatient care in pediatric wards	18	He told us to consult a psychiatrist. Of course I was not willing to do so because my daughter was still very young. He saw that I didn’t want to, so he asked me if it was possible to let my daughter stay home… and not to force her to eat. I said no. I wouldn’t have to come to the accident and emergency department if I could, right? Afterwards, he said he could transfer my daughter to the pediatric ward and asked if I was willing to take her there. I found this more acceptable so we went to seek help there. (P1)
Assistance from patient support organizations	19	We obtained a lot of information from the eating disorder association, and parents at the association gave us a lot of suggestions on how we should handle certain scenarios during our children’s recovery process. (P3)
Supportive school environment	20	Both the principal and the vice-principal were very nice. They told me that my daughter probably had anorexia when they saw how skinny she was. They immediately advised me to visit the eating disorder association and meet Dr. X (a professor specialized in eating disorders)… They also allowed my daughter to skip her final exams. They assured us that she would be promoted to Form 3 as long as she has recovered. (P4)
	21	The vice-principal and the school social worker were very caring. They often communicated with us on phone and would have special arrangements for my daughter regarding her studies. This gave my daughter greater flexibility and she had more choices. (P3)
Empathetic health professionals	22	The psychiatrist was clever, and she found what he said acceptable. Therefore, she was willing to see him. She wants other people to respect her and be able to communicate well with her. (P4)
Management approach of parents	23	Actually, it is futile to consult psychiatrists. Unlike other common diseases, there are no specific medication or procedure like surgery that can cure the disease. To a certain extent, the family has to take up the role [in patient management]. Perhaps family members have to change [their parenting styles] in order to make her… it would help her get better, I guess. (P5)
	24	At first, my daughter felt that it was alright and was willing to eat. Maybe I was too impatient, I pushed her further and asked her to eat a relatively big piece of fish. Perhaps I was too impatient and I have gone too far. (P1)
	25	I was very strict with my children. I believed I was right as my son was able to withstand it, and I thought I should also be strict with my daughter. However, my daughter couldn’t stand it and she even became more rebellious. I realize that I can’t just go by the book and I shouldn’t be strict with them. (P6)
The need for psychological support	26	Both of our daughters were suffering from anorexia, and people around them were extremely anxious about it… We felt very tired and disappointed most of the time. There was nothing we could do after trying 10 different types of treatments. (P2)
	27	Let me explain it this way, as parents, we are looking for something that offers more psychological support than what psychologists can offer… I think this is more like treating the parents. This is more important than anything else, as parents care about their children the most. If parents could be effectively treated, their children would also have a higher chance of rapid recovery. I think this would be more important. (P3)

#### Inpatient care in pediatric wards

Some parents avoided sending their children to psychiatric hospitals because of their young age and public stigma. They were more willing to have their children treated at the pediatrics department (Table 3, Quote 18).

#### Assistance from patient support organizations

P2 pointed out that despite he did not seek as much help as the other participants, his child had attained the best outcomes. Learning from the stories of other parents in the eating disorder association was the best help he ever got. He also appreciated how parents at the association offered mutual support to one another (Quote 19).

#### Supportive school environment

The support from school was identified as an important enabler. Some parents were advised by their children’s school to seek help. The children got encouragement and special arrangement such as exam exemption during the treatment process (Quotes 20 and 21).

#### Empathetic health professionals

Empathy from health professionals helped to build up trust between the patient and the doctor, and might enhance treatment intention and follow-up care (Quote 22).

### Reflections from parents

#### Management approach of parents

As professional help alone was perceived as ineffective, parents gradually realized their significant role in contributing to their children’s recovery (Quote 23). Some parents also reflected on the problems with their management approaches. P1 thought she might be too impatient (Quote 24), and P6 thought she had to modify her authoritarian parenting style (Quote 25).

#### The need for psychological support

Parents were generally stressed and did not know how they should take care of their children with AN. P2 was in despair because he had tried various types of treatment with disappointing outcomes (Quote 26). P3 expressed her need for psychological support and believed that sufficient support for parents was crucial in the child’s recovery (Quote 27).

## Discussion

The interviews revealed the dissatisfaction of the parents with the management of AN by health professionals. Many of them had limited trust in the health professionals, in aspects of both knowledge and attitude. While current FBT models of AN recognize parental and family factors as important causes of AN, the treatment aims to externalize and depersonalize the disorder [21]. Thus, rather than attributing the cause of AN to any particular family member, the whole family is encouraged to fight against their common foe–AN. The psychiatrist’s criticism perceived by P1 on her parental approach indicated that this treatment concept had not been endorsed by some psychiatrists. In fact, the parents were also unclear about the treatment model as they received little explanation from the health professionals. Even though their children might be undergoing phase 1 of FBT for weight restoration at home, the parents did not understand well the meaning of their role. P1 found it difficult to monitor her daughter’s home diet, and felt the psychiatrist had shifted the treatment responsibility to her. The treatment outcome could have been greatly enhanced if a clear concept of the treatment approach was offered to the parents and reinforced throughout the process by means of psychoeducation. It is also important for the psychiatrists to have updated knowledge about FBT and other common models. The spirit of FBT is not only about family involvement but also family empowerment. Similar concepts of empowerment should also be applied to treatment for those admitted to the hospital since both parents and their children shared the fear of being hospitalized.

The qualitative quotes also revealed the high standards demanded by the parents, not only about treatment outcomes, but also communication styles of the health professionals. Similar feedbacks were reported from the parents of AN adolescents in a UK qualitative study in which the parents’ narratives conveyed a sense of “hoping for the best, but fearing the worst” [37]. Other Western studies also showed that the parents felt being misunderstood, scrutinized and blamed during their interaction with the health professionals [38]. The major criticisms of the parents in our study were about non-supportive attitudes by health professionals and inadequate communication or improper management of caregivers’ expectations. While it is essential to improve the health care services, such as more empathy to the patients and carers, higher quality of follow-up consultations and detailed explanations of treatment plan, it is often unavoidable for patients to compromise to a certain extent in a non-ideal health care setting. For instance, P6 could remind the clinical psychologist if there were certain aspects that he/she had not followed up in subsequent visits. In fact, internationally the recovery rate of AN is not high in the short term. It could be as low as 29% after 2.5 years of follow up in a Demark study [39], but increased to around 57–67% after 4–5 years of follow up in studies from Finland, the Netherlands and the US [40–42]. In terms of mortality rate, favorable results were reported in a Sweden study for a representative sample of subjects after 18 years of teenage onset, in contrast to the high mortality rate of 10–20% found among severe in-patient cases in previous studies [14]. A pragmatic management goal is to stabilize the disorder so that the condition is not life threatening. Increasing the patients’ weight has been the top priority [13, 22]. Nonetheless, this was perceived by the parents of our study as a shortcoming that the treatment only dealt with weight restoration. The same critique had also been reported by other Western studies [26, 27]. In a deeper sense, limited psychoeducation and communication regarding the treatment goal and process may be a bigger problem in a Chinese context as reflected from the parents’ experience. More genuine communication between the health professionals and the parents about the expected treatment outcomes and limitations may enhance their mutual trust, which has been identified as a crucial facilitator in treating AN [30]. Moreover, patients in Asian countries may encounter more difficulties in finding a preferred professional to treat AN. While the parents in our study generally perceived the professionals involved as unhelpful, all parents in a UK study turned out being able to find a professional who they could call on for help [37]. Another study, conducted in Norway, reported that the parents were generally positive about the therapists, including both knowledge and communication approach [43].

The parents in our study also perceived insufficient coordination among the health professionals. Some said that they needed to take up the role as a coordinator. Without professional clinical knowledge, it was difficult for the parents to integrate the information given by different health professionals. The situation would be better if their family doctors could help them to digest the medical information, coordinate with other health professionals, and to provide psychosocial support when the parents are frustrated during the long treatment process. A recent study in Hong Kong showed that patients with a regular GP had a double probability to be managed for psychological issues compared with the other patients [44]. Although the GPs are not expected to have expertise knowledge in treating AN, a basic understanding of AN and the FBT model is needed. Similarly, the social workers in schools or hospitals also require knowledge of AN. The advice from the medical social worker to P5 about stopping her daughter to have snacks was like a casual suggestion by a layman. In opposite, the school social worker contributed to the identification of AN and recovery of the daughter of P3. Thus, apart from the specialists, the primary healthcare professionals can also be significantly involved in the management of AN.

This study has several limitations. First, only six parents with AN children were interviewed as its prevalence is not high compared with other mental disorders. A systematic review showed that the usual number of parents interviewed in qualitative studies on ED was around 5–20 [38]. Our participants were recruited among the members of an eating disorder association and might reflect the situations of the difficult-to-treat cases. Second, we interviewed the parents rather than their children due to ethical considerations and practical difficulties in recruiting the latter. The views of the parents revealed their feelings as a carer giver while the children’s experiences might uncover another side of the story. Third, we focused on the help seeking, treatment process and outcomes of the cases without considering the effects of the children’s co-morbid physical and psychological conditions on AN. Finally, we did not ask about their children’s exact AN onset age and stage of adolescence. Most cases progressed for a long period during adolescence, and some reached young adulthood.

## Conclusions

Limited psychoeducation, communication and coordination in the treatment for AN are major problems in a Chinese context. Insufficient communication between the health professionals and the parents of AN adolescents creates tension in their relationship and affect the management outcomes. The core concepts of the treatment model like FBT should be explained clearly to the parents so they can better understand their role. Besides, the health professionals should keep in mind that FBT is not only about family involvement but also family empowerment. More genuine communication about the expected treatment outcomes and limitations are essential to improve their mutual trust.
